# Gastrointestinal microbiota and metabolites responses to dietary cereal grains in an adult pig model

**DOI:** 10.3389/fmicb.2024.1442077

**Published:** 2024-09-17

**Authors:** Ganyi Feng, Menglong Deng, Rui Li, Gaifeng Hou, Qing Ouyang, Xianji Jiang, Xiaojie Liu, Hui Tang, Fengming Chen, Shihua Pu, Dan Wan, Yulong Yin

**Affiliations:** ^1^Key Laboratory of Agro-Ecological Processes in Subtropical Region, Hunan Provincial Key Laboratory of Animal Nutritional Physiology and Metabolic Process, Hunan Research Center of Livestock and Poultry Sciences, South Central Experimental Station of Animal Nutrition and Feed Science in the Ministry of Agriculture, National Engineering Laboratory for Poultry Breeding Pollution Control and Resource Technology, Institute of Subtropical Agriculture, Chinese Academy of Sciences, Changsha, China; ^2^College of Animal Science and Technology, Hunan Co-Innovation Center of Animal Production Safety, Hunan Agricultural University, Changsha, China; ^3^Hunan Provincial Key Laboratory of the TCM Agricultural Biogenomics, Changsha Medical University, Changsha, China; ^4^Chongqing Academy of Animal Science, Rongchang, Chongqing, China; ^5^National Center of Technology Innovation for Pigs, Chongqing, China

**Keywords:** corn, wheat, paddy rice, GIT, microbial community, SCFAs

## Abstract

Corn (C), wheat (W), and paddy rice (PR) are important energy sources and are commonly used in feed production for swine. This study mainly focuses on the variation and regularities of microbiota and metabolites in the gastrointestinal tract (GIT) of pigs in response to C, W, and PR. A total of 18 pigs were allotted into three dietary groups with six replicated pigs and received diets containing C, W, or PR as the sole energy source, respectively. The results showed that digestive parts significantly affected the diversity of microbial communities. Cereal grain sources significantly influenced the β-diversity of microbial communities in the colon and rectum. Campylobacterota and Proteobacteria are mainly distributed in the duodenum, *Lactobacillus* in the jejunum, and Bacteroidota in the colon and rectum. The W diet increased the Bacteroidota, Spirochaetota, and *Prevotellaceae_NK3B31_group* abundances and showed the highest concentrations of all short-chain fatty acids (SCFAs) in the hindgut. Fibrobacterota, Bacteroidota, Spirochaetota, *Prevotellaceae_NK3B31_group*, *Prevotella*, and *Treponema* in the colon or rectum were positively correlated with acetate, propionate, butyrate, and total SCFAs. These findings suggested that aerobic bacteria and facultative anaerobes in the foregut will gradually be replaced by anaerobes in the hindgut. The W diet had the best fermentability and was beneficial to the colonization of microbial communities that mainly used carbohydrates. The hindgut flora of the PR diet group may be more balanced with fewer potential pathogenic bacteria. Many microbial communities have been identified to contribute positively to the SCFA production of the hindgut. Collectively, our study revealed the spatial variation regularities of GIT microbial communities in an adult pig model and provided new insights into GIT microbiota and responses of metabolites to cereal grain diets.

## 1 Introduction

Cereal grains are the most important energy component in the diet of monogastric animals and are widely used in the feed industry. Cereal grains contain easily digestible starch and high-quality plant-based protein (Singh et al., 2010; Gidley, 2023). Although resistant starch (RS) and dietary fibers (DFs) in cereal grains cannot be digested and absorbed by the stomach and small intestine, they will be fermented by microorganisms in the large intestine to produce SCFAs (Jaworski and Stein, 2015; Rodehutscord et al., 2016; Holscher, 2017; Walsh et al., 2022; Gidley, 2023). The physicochemical properties and chemical composition of different cereal grains vary considerably, especially the composition of DF (Cervantes-Pahm et al., 2014; Tan et al., 2021). Fermentable DF from different cereal grains may regulate the microbial community composition in the host GIT and affect the metabolism of SCFAs in the hindgut (O'Connell et al., 2005; Wong et al., 2006; Van der Meulen and Jansman, 2010; Liu et al., 2012; Ma et al., 2018; Zhao et al., 2019). A previous study found that a barley-based diet increased the ratio of *Lactobacillus* spp. to *Enterobacteriaceae* in ileal digesta and fecal samples of pigs, while a W-based diet promoted the proliferation of *Roseburia* spp. in the ileum and *Bifidobacterium* spp. in the fecal samples (Weiss et al., 2016). Zhao et al. (2019) reported that different DF influenced the abundances of *Filifactor* and *Intestinibacter* in ileal digesta, as well as *Ruminococcus_1* and *Lachnoclostridium* in the feces of pigs. In addition, the production of SCFAs in ileal digesta and feces was associated with microbial compositions.

The GIT microbiota is a huge, dynamic, and complex ecosystem and plays an important role in nutrient digestion, absorption, and metabolism, as well as in the maintenance of intestinal health (Lallès, 2016; Holman et al., 2017; Tan et al., 2018). Fully understanding the dynamic distribution of the gut microbiota in pigs is essential (Luo et al., 2022). At present, although there are many studies on the composition and distribution of intestinal microbiota in pigs, most of them selected colonic or fecal samples with more abundant microorganisms (O'Hara and Shanahan, 2006; Gao et al., 2019a; Gao et al., 2019b; Li et al., 2019a; Li et al., 2019b; Li et al., 2023). Few studies have focused on the whole-gut microbiota of pigs, which limits a comprehensive understanding of the spatial variation and regularities of the GIT microbiota in swine.

Zhao et al. (2015) highlighted that although the composition and structure of the microbial community become stable when the host is mature, and there are significant similarities between feces and the large intestine microbiome, using fecal samples alone cannot fully represent the microbial profile of the GIT. Therefore, observing the dynamic variation of microorganisms along the GIT is a necessary consideration when studying the interaction between hosts and microorganisms. In recent years, some researchers have explored and summarized the characteristics and dynamic distribution of gut microbiota across different ages and GIT segments of pigs (Zhao et al., 2015; Liu et al., 2019; Luo et al., 2022; Fabà et al., 2024). However, it is worth noting that these studies have selected young pigs (newborn piglets and nursery pigs) as models. There have been no relevant reports on the dynamic distribution of the GIT microbiota in adult pigs (especially for finishing pigs with heavier weight) models, which is not conducive to enriching the scientific community’s understanding of the gut microbiome dynamics for swine.

C, W, and PR were the three important food crops in terms of total production around the world (FAOSTAT, 2023). They are also widely used in pig’s diets. However, few studies have investigated the effect of these cereal grains on the GIT microbiota and metabolites. Therefore, this study aimed to characterize the microbial composition of adult pigs from the stomach to the rectum and to investigate the effects of C, W, and PR on the composition and SCFA production of the GIT microbiota.

## 2 Materials and methods

### 2.1 Animals, diets, and experimental design

**Figure 1:**
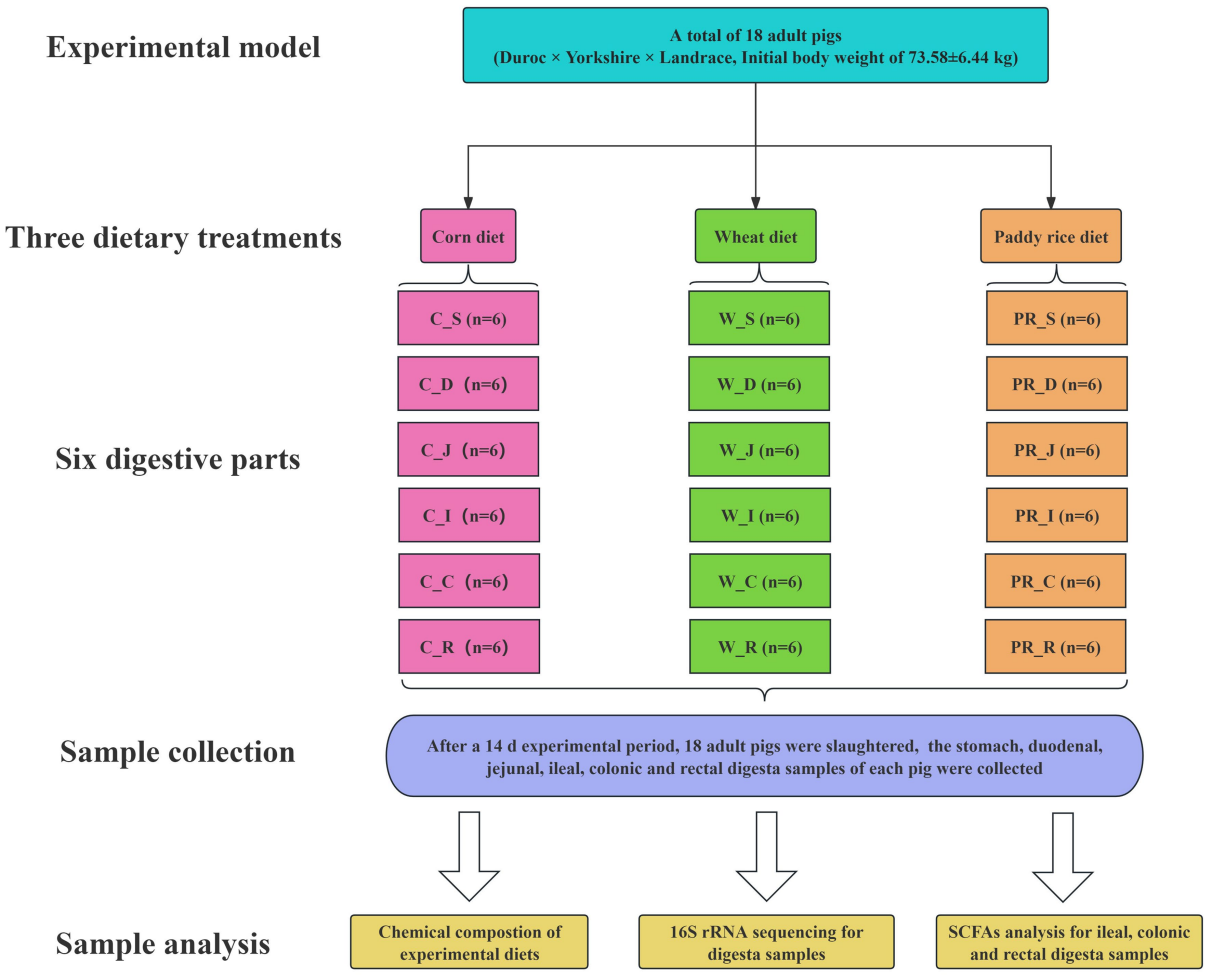
Flow diagram of the experimental design. C, corn; W, wheat; PR, paddy rice; S, stomach; D, duodenum; J, jejunum; I, ileum; C, colon; R, rectum.

In total, 18 pigs (Duroc × Yorkshire × Landrace, initial body weight of 73.58 ± 6.44 kg) fitted with a simple T-cannula in their distal ileum were allotted to three dietary treatments with six replicated pigs (Figure 1). During the 14-day experimental period, pigs were fed diets with C, W, and PR as the sole energy source, respectively (Table 1). Vitamins and minerals were supplemented to meet or exceed the nutrient requirements recommended by the NRC (2012). Pigs were placed in individual metabolism crates (1.4 m × 0.7 m × 0.5 m) and kept in an environmentally controlled room (23 ± 1°C). From days 1 to 14, the daily feeding schedule was divided into two equal meals provided at 0830 and 1730 h, respectively. The daily feeding amount of each pig was fixed at 4% of the average initial weight of pigs (Adeola, 2000). Each pig had free access to water via a nipple drinker.

**Table 1:** Ingredient and analyzed chemical compositions of diets (as-fed basis, %).

Items	Corn diet	Wheat diet	Paddy rice diet
Corn	97.00	-	-
Wheat	-	97.00	-
Paddy rice	-	-	97.00
Dicalcium phosphate	1.00	1.00	1.00
Limestone	0.90	0.90	0.90
Sodium chloride	0.30	0.30	0.30
Titanium dioxide	0.30	0.30	0.30
Vitamin and mineral premix^1^	0.50	0.50	0.50
Total	100.00	100.00	100.00
Analyzed composition			
DM	88.53	90.23	88.98
CP	8.44	11.12	7.57
GE, MJ/kg	14.51	13.21	14.51
EE	0.86	1.80	1.89
Ash	3.08	4.42	6.29
NDF	25.21	17.75	22.89
ADF	2.41	3.02	13.93
CF	1.26	1.59	8.24
IDF	8.92	10.21	17.44
SDF	1.16	1.37	0.83
TDF	10.08	11.58	18.27
TS	56.22	46.21	56.88

DM, dry matter; CP, crude protein; GE, gross energy; EE, ether extract; NDF, neutral detergent fiber; ADF, acid detergent fiber; CF, crude fiber; IDF; insoluble dietary fiber; SDF; soluble dietary fiber; TDF, total dietary fiber; TS, total starch.

1 The premix provided the following per kg of diets: Vitamin A 4,200 IU, VD_3_ 400 IU, VE 36 IU, VK_3_ 1.2 mg, VB_12_ 23 μg, VB_2_ 5.63 mg, VB_5_ 20.5 mg, VB_3_ 28 mg, Choline chloride 1.00 g, Folic acid 0.8 mg, VB_1_ 3.4 mg, VB_6_ 2.7 mg, VB_7_ 0.18 mg, Mn (as manganese sulfate) 40.0 mg, Fe (as ferrous sulfate) 70.0 mg, Zn (as zinc sulfate) 70.0 mg, Cu (as copper sulfate) 70 mg, I (as potassium iodide) 0.3 mg, and Se (as sodium selenite) 0.3 mg.

### 2.2 Sample collection

From 0800 h on 15th d, all pigs were slaughtered, the GIT was removed immediately, and the stomach (S), duodenum (D), jejunum (J), ileum (I), colon (C), and rectum (R) were carefully identified, separated, and ligated with a thin thread. While ensuring that digesta did not move as much as possible, a scissor was used to cut open each digestive part, and digesta from six digestive parts was collected as much as possible using numbered 50 ml centrifuge tubes or beakers with larger volumes. After stirring evenly with a glass rod, four representative samples of the stomach, duodenal, jejunal, ileal, colonic, and rectal digesta from each pig were collected using a 1.5 ml centrifuge tube. These samples were divided into 18 groups (*n* = 6) according to the corresponding digestive part and dietary treatment. Each group was named according to the form “cereal grain abbreviation_digestive part abbreviation” (e.g., C_S group). These samples were first snap-frozen in liquid nitrogen and stored in a −80°C refrigerator at the end of sampling. One digesta sample per pig from 18 groups was selected for 16S rRNA gene sequence analysis (*n* = 108). One additional digesta sample from each of the ileum, colon, and rectum was selected for SCFA analysis (*n* = 54).

### 2.3 Chemical composition analysis of experiment diets

The C diet, W diet, and PR diet were finely ground to pass through a 1-mm screen. The dry matter (DM, 930.15), ether extract (EE, 920.39), and ash (942.05) contents of diets were analyzed using the Association Official Analytical Chemists (AOAC, 2012). The crude protein (CP) content of diets was determined by the various MAX cube Carbon/Nitrogen/Sulfur Elemental Analyzer (Elementar Analysensysteme GmbH, Frankfurt, Hessian, Germany). The content of gross energy (GE) in diets was determined by an HXR-6000 Oxygen Bomb Automatic Calorimeter (Hunan Huaxing Energy Sources Instrument Co. Ltd., Changsha, China). The contents of crude fiber (*CF*), neutral detergent fiber (NDF), and acid detergent fiber (ADF) were determined using fiber bags and the FT12 automatic fiber analyzer (Gerhardt Analytical Instrument Co., Ltd., Konigswinter, North Rhine-Westphalia, Germany) by the basic procedures of Van Soest et al. (1991). The contents of soluble dietary fiber (SDF) and insoluble dietary fiber (IDF) were analyzed according to the procedure described in a commercial reagent kit (K-TDFR-200A, Megazyme Inc., Wicklow, Ireland). Total starch (TS) was measured according to the AOAC Method 996.11 with the K-TSTA TS assay kit (Megazyme Inc., Wicklow, Ireland).

### 2.4 SCFA analysis

The SCFA concentrations in the ileal, colonic, and rectal digesta samples were analyzed according to the method described by Wang et al. (2017). Briefly, approximately 150 mg samples were weighed into a 1.5 ml centrifuge tube, then diluted with 450 μl of deionized water, including 1.68 mM heptanoic acid/L as an internal standard (Sigma Chemical Co., St. Louis, MO, USA). The samples were centrifuged at 10,000 rpm for 10 min at 4°C after thoroughly mixing with the OSE-Y50 high-speed grinder (Tiangen Biochemical Technology Co., Ltd., Beijing, China), and the supernatant was all absorbed and filtered with a 0.22 μm membrane. The acetate, propionate, isobutyrate, butyrate, isovalerate, valerate, and total SCFAs were measured and quantified using the 7820A gas chromatography (GC) system (Agilent Technologies, Palo Alto, CA, USA).

### 2.5 DNA extraction, GIT microbes, and 16S rRNA sequencing

Total microbial genomic DNA in digesta (the stomach, duodenum, jejunum, ileum, colon, and rectum) was extracted using the E.Z.N.A.^®^ Soil DNA Kit (Omega Bio-Tek Inc., Norcross, GA, USA). The quality and concentration of DNA were detected by 1.0% agarose gel electrophoresis and the NanoDrop^®^ ND-2000 spectrophotometer, respectively (Thermo Scientific Inc., Boston, MA, USA). The hypervariable V3–V4 region of the bacterial 16S rRNA gene was amplified with primer pairs 338F (5′-ACTCCTACG GGAGGCAGCAG-3′) and 806R (5′-GGACTACHVGGTWT CTAAT-3′) using the ABI GeneAmp^®^ 9700 PCR thermocycler (Applied Biosystems Inc., Los Angeles, CA, USA; Liu et al., 2016). PCR was carried out in triplicate using 20 μl reactions containing 10 ng of template DNA, 4 μl of 5 × Fast Pfu buffer, 2 μl of dNTPs, 0.8 μl of each primer, 0.4 μl of Fast Pfu polymerase, 0.2 μl of BSA, and 12.6 μl of ddH_2_O. The PCR product was extracted from 2% agarose gel, purified using the AxyPrep DNA Gel Extraction Kit (Axygen Biosciences, Union City, CA, USA), and quantified using the Quantus™ fluorometer (Promega Inc., Madison, WI, USA). Purified amplicons were pooled in equimolar amounts and paired-end sequenced on an Illumina MiSeq PE300 platform/NovaSeq PE250 platform (Illumina Inc., San Diego, CA, USA).

### 2.6 Bioinformatics analysis

Raw sequence data were quality-filtered and merged using Fastp (version 0.19.6) and Flash (version 1.2.11) software, respectively (Magoč and Salzberg, 2011; Chen et al., 2018). The optimized sequences were clustered into operational taxonomic units (OTUs) using Uparse software with a 97% sequence similarity level (Stackebrandt and Goebel, 1994; Edgar, 2013). The number of 16S rRNA gene sequences from each sample was rarefied to 66,859, and the average coverage index of samples was 99.66% after rarefying. OTUs representing <0.005% of the population were removed, and taxonomy was assigned using the RDP classifier (Wang et al., 2007; Li et al., 2023). R Programming Language (version 3.3.1) was used to generate Venn diagrams to visualize the occurrence of shared and unique OTUs among groups and to make pie and bar plots to represent the relative abundance of taxonomic groups on the phylum and genus levels.

The Mothur software (version 1.30.2) was used for calculating the Shannon, Ace, Simpson, and Chao1 indices on the OTU level (Schloss et al., 2009; Edgar, 2013). The principal coordinate analysis (PCoA) based on the Bray–Curtis dissimilarity was performed to determine the similarity among the microbial communities in different groups (Li et al., 2021). Analysis of similarities (ANOSIM) was performed to compare the difference in microbial communities at six digestive parts and three dietary treatments. The significantly abundant bacterial taxa from the phylum to genus level among the different groups (LDA score > 2, *p* < 0.05) were identified using the linear discriminant analysis (LDA) effect size (LEfSe) software (Wang et al., 2007; Segata et al., 2011). The pheatmap package of R programming language (version 3.3.1) was used to calculate Spearman’s coefficient and draw the correlation heatmap.

### 2.7 Statistical analysis

GraphPad Prism (version 10.1.2, La Jolla, CA, USA) and IBM SPSS Statistics (version 22.0, IBM, Chicago, IL, USA) software were used for plotting graphs and statistical analysis. For Shannon, Simpson, Ace, and Chao1 index data, significant differences between treatments were assessed by two-way ANOVA using Turkey’s multiple comparisons test with each pig as an experimental unit, and digestive parts and treatment diet were two fixed effects, considering their interaction. For SCFA concentration data from the ileum to the rectum, significant differences between treatments were assessed by one-way ANOVA using Duncan’s multiple comparisons test with each pig as an experimental unit, and treatment diet was the only fixed effect. For data on the relative abundances of microbial communities, the Kruskal–Wallis and Tukey–Kramer tests were used to evaluate the statistical significance among the multiple groups. Statistical significance was declared at a *p*-value of <0.05.

## 3 Results

### 3.1 Tag- and OTU-based analysis

A total of 25,880,835 sequences passed the quality control, with an average of 239,637 high-quality sequences per sample and an average sequence length of 416 bp. As shown in Supplementary Figure 1, the Venn analysis identified 1,497, 1,302, 1,091, 1,120, 3,076, and 2,972 unique OTUs in the stomach, duodenum, jejunum, ileum, colon, and rectum groups, respectively.

### 3.2 The diversity of bacterial communities in samples

**Figure 2:**
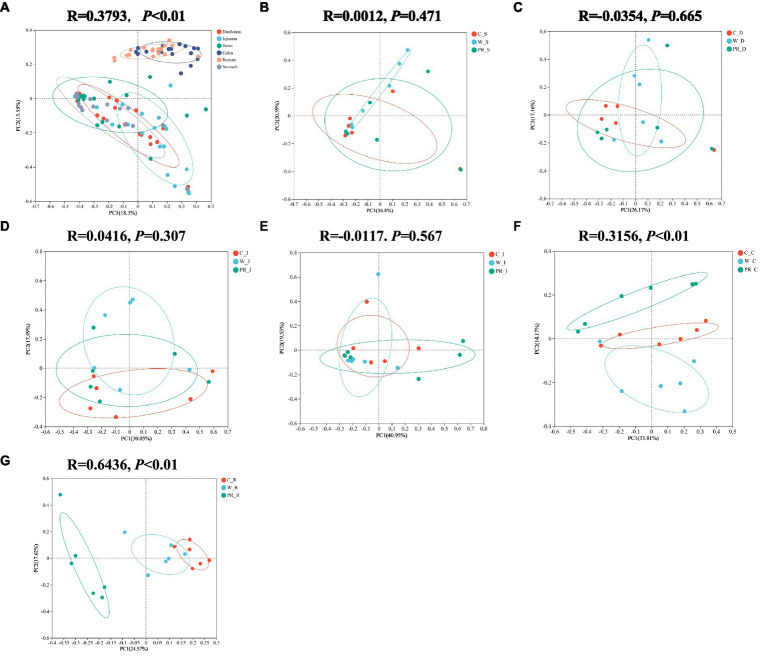
Principal coordinates analysis (PcoA) of microbial composition in six digestive parts **(A)**. PcoA of microbial composition in the stomach **(B)**, duodenal **(C)**, jejunal **(D)**, ileal **(E)**, colonic **(F)**, and rectal **(G)** digesta of adult pigs fed with different cereal diets. C, corn; W, wheat; PR, paddy rice; S, stomach; D, duodenum; J, jejunum; I, ileum; C, colon; R, rectum.

As shown in Table 2, non-dietary grain sources but digestive parts significantly affect the α-diversity indices of microbial communities (*p* < 0.01). The interaction effect of diets and digestive parts had a significant effect on Simpson and Ace indices (*p* < 0.05). The microbial community in the colon and rectum groups mostly showed higher Shannon, Ace, Chao1, and lower Simpson indices than other groups (*p* < 0.01 or *p* < 0.05, Table 2 and Supplementary Figures 2, 3). The results of PCoA based on Bray–Curtis distance and evaluated by the ANOSIM test indicated significant differences in microbial communities in the colon and rectum compared to other digestive parts (Figure 2A, *p* < 0.01) There are no significant differences in the stomach, duodenal, jejunal and ileal microbiota among three dietary treatments (Figures 2BE). There are significant differences in the colonic and rectal microbiota among three dietary treatments (Figures 2F,G, *p* < 0.01).

**Table 2:** Effects of grain diets on intestinal microbial α-diversity indexes of adult pigs.

Treatment	Digestive parts	Shannon index	Simpson index	Ace index	Chao1 index
Corn diet	Stomach	1.22 ± 0.57^b^	0.44 ± 0.15^a^	292.04 ± 86.88^b^	279.52 ± 103.21^b^
	Duodenum	1.71 ± 1.01^b^	0.28 ± 0.14^ab^	367.14 ± 215.32^b^	368.68 ± 222.13^b^
	Jejunum	1.56 ± 0.96^b^	0.39 ± 0.23^a^	370.31 ± 130.37^b^	373.00 ± 132.86^b^
	Ileum	1.83 ± 0.32^b^	0.25 ± 0.07^abc^	212.79 ± 132.26^b^	170.47 ± 64.65^b^
	Colon	4.30 ± 0.67^a^	0.08 ± 0.09^bc^	2361.40 ± 332.38^a^	1994.75 ± 219.05^a^
	Rectum	4.79 ± 0.17^a^	0.02 ± 0.00^c^	2156.24 ± 325.48^a^	1967.71 ± 172.00^a^
Wheat diet	Stomach	2.26 ± 0.86^b^	0.25 ± 0.15^ab^	567.11 ± 459.52^b^	557.87 ± 442.03^bc^
	Duodenum	2.39 ± 1.23^b^	0.28 ± 0.22^ab^	676.40 ± 466.45^b^	660.97 ± 470.59^b^
	Jejunum	1.95 ± 1.11^b^	0.38 ± 0.25^a^	359.67 ± 112.32^b^	342.02 ± 114.82^bc^
	Ileum	1.72 ± 0.32^b^	0.28 ± 0.12^ab^	222.82 ± 110.35^b^	163.41 ± 63.38^c^
	Colon	4.24 ± 0.57^a^	0.09 ± 0.08^b^	2051.29 ± 354.80^a^	1992.74 ± 339.20^a^
	Rectum	4.57 ± 0.19^a^	0.04 ± 0.01^b^	2165.32 ± 257.56^a^	2091.11 ± 197.56^a^
Paddy rice diet	Stomach	2.07 ± 1.22^c^	0.21 ± 0.11	564.66 ± 337.37^b^	576.81 ± 345.22^b^
	Duodenum	1.93 ± 1.09^c^	0.22 ± 0.14	535.92 ± 150.98^b^	481.70 ± 131.46^b^
	Jejunum	2.25 ± 1.14^bc^	0.21 ± 0.10	587.70 ± 228.86^b^	560.71 ± 227.45^b^
	Ileum	1.76 ± 0.75^c^	0.32 ± 0.10	471.79 ± 449.22^b^	420.24 ± 449.22^b^
	Colon	3.60 ± 1.28^ab^	0.21 ± 0.24	1864.45 ± 363.87^a^	1197.63 ± 359.39^a^
	Rectum	3.90 ± 0.67^a^	0.12 ± 0.13	1961.49 ± 163.62^a^	1911.61 ± 145.90^a^
Corn diet					
Wheat diet					
Paddy rice diet					
	Stomach	1.85 ± 0.99^b^	0.30 ± 0.16^a^	474.60 ± 339.78^b^	471.40 ± 339.42^b^
	Duodenum	2.01 ± 1.08^b^	0.26 ± 0.16^ab^	526.48 ± 318.21^b^	503.78 ± 316.33^b^
	Jejunum	1.92 ± 1.04^b^	0.33 ± 0.21^a^	439.22 ± 189.22^b^	425.24 ± 184.85^b^
	Ileum	1.77 ± 0.48^b^	0.28 ± 0.10^a^	302.46 ± 288.58^b^	251.37 ± 277.25^b^
	Colon	4.04 ± 0.90^a^	0.13 ± 0.16^bc^	2092.38 ± 391.07^a^	1928.37 ± 308.20^a^
	Rectum	4.42 ± 0.55^a^	0.06 ± 0.08^c^	2094.34 ± 260.58^a^	1990.14 ± 179.98^a^
*p*-value	Diets	0.287	0.636	0.765	0.167
	Digestive parts	<0.01	<0.01	<0.01	<0.01
	Diets × Digestive parts	0.290	0.047	0.019	0.187

All data were analyzed by the Kruskal-Wallis and Tukey-Kramer test. The results were presented as mean values ± standard deviation (*n* = 6 per treatment). Means in each column with different letters differs significantly (*p* < 0.05).

### 3.3 Composition and differential analysis of microbial communities among six digestive parts

**Figure 3:**
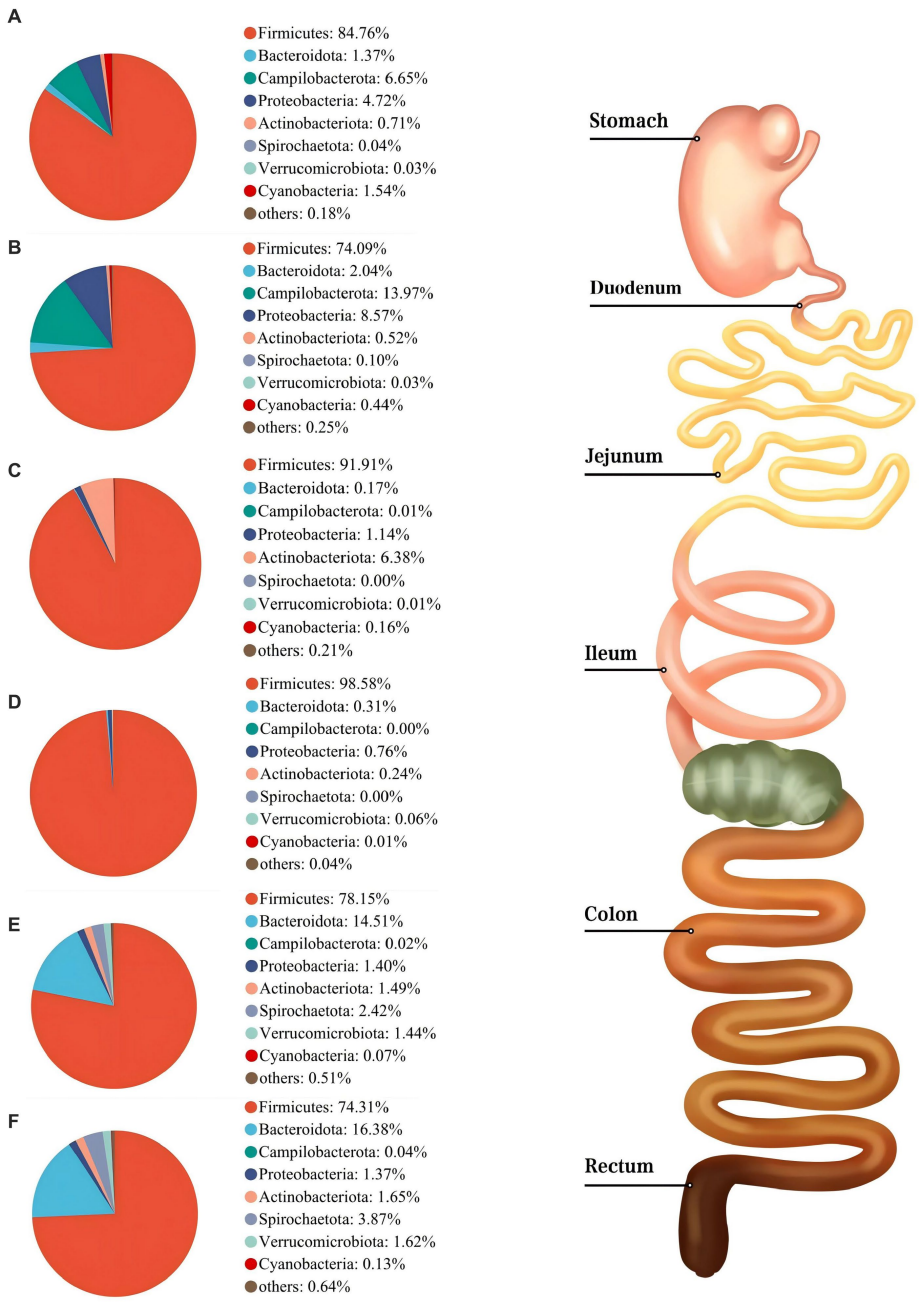
Composition of microbiota in the stomach **(A)**, duodenum **(B)**, jejunum **(C)**, ileum **(D)**, colon **(E)**, and rectum **(F)** based on the phylum level.

**Figure 4:**
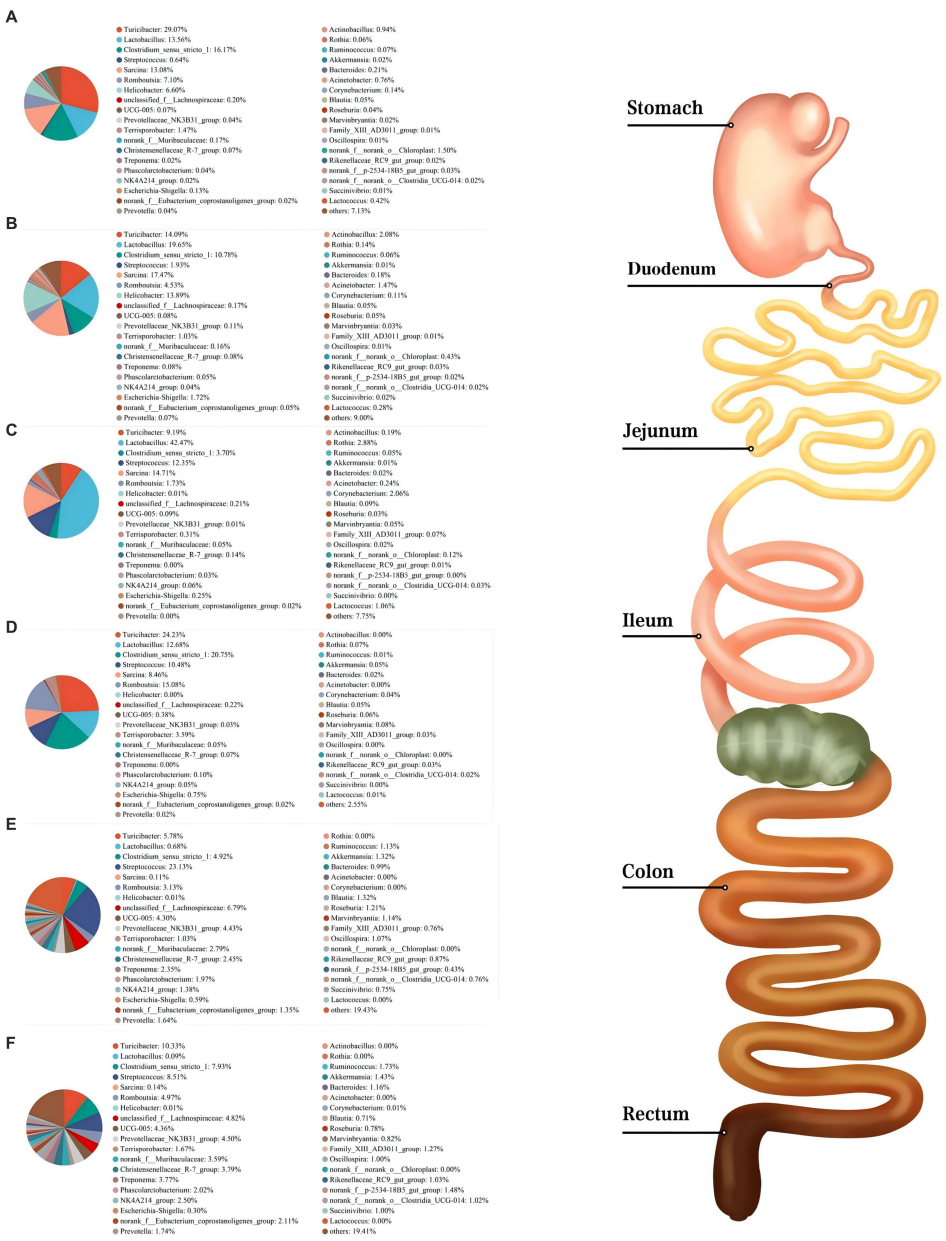
Composition of microbiota in the stomach **(A)**, duodenum **(B)**, jejunum **(C)**, ileum **(D)**, colon **(E)**, and rectum **(F)** based on the genus level.

**Figure 5:**
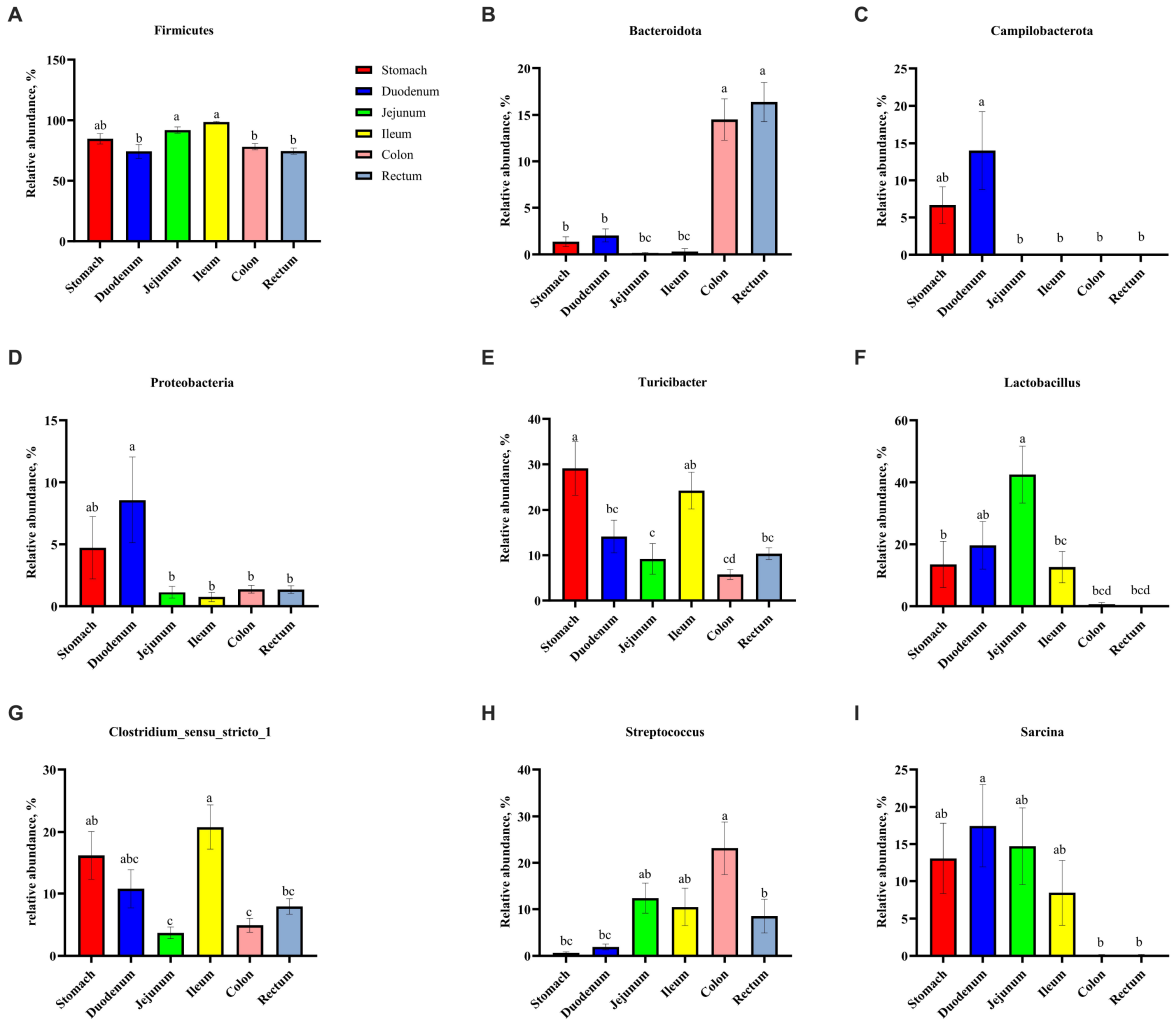
Comparative analysis of microbiota among six digestive parts based on the phylum **(A–D)** and the genus **(E–I)** levels. All data were analyzed by the Kruskal–Wallis and Tukey–Kramer test and presented as the mean percentage of different bacteria (group = 6, *n* = 18 per treatment). The different letters mean *p* < 0.05.

As shown in Figure 3, Firmicutes was the most dominant phylum among six digestive parts, its relative abundance was 84.76% (the stomach), 74.09% (the duodenum), 91.91% (the jejunum), 98.58% (the ileum), 78.15% (the colon), and 74.31% (the rectum), respectively. Bacteroidota is another dominant phylum in the colon and rectum, with relative abundance of 14.51% (colon) and 16.38% (rectum), respectively. *Turicibacter*, *Lactobacillus*, and *Clostridium_sensu_stricto_1* were the dominant genera in the stomach, duodenum, jejunum and ileum. *Streptococcus* (23.13%) was the most dominant genus in the colon, whereas *Turicibacter* (10.33%) was dominant in the rectum (Figure 4). The jejunum and ileum groups had a high abundance of Firmicutes (*p* < 0.05, Figure 5A). The colon and rectum groups showed greater Bacteroidota abundance than other groups (*p* < 0.05, Figure 5B). The populations of Campylobacterota, Proteobacteria, and *Sarcina* were largest in the duodenum group (*p* < 0.05, Figures 5C,D,I). *Lactobacillus* in the jejunum and *Streptococcus* in the colon had the highest relative abundance, respectively (*p* < 0.05, Figures 5F,H). The ileum group showed higher relative abundances of *Turicibacter* and *Clostridium_sensu_stricto_1* than the jejunum and colon groups (*p* < 0.05, Figures 5E,G).

### 3.4 Composition and differential analysis of microbial communities among dietary treatments

**Figure 6:**
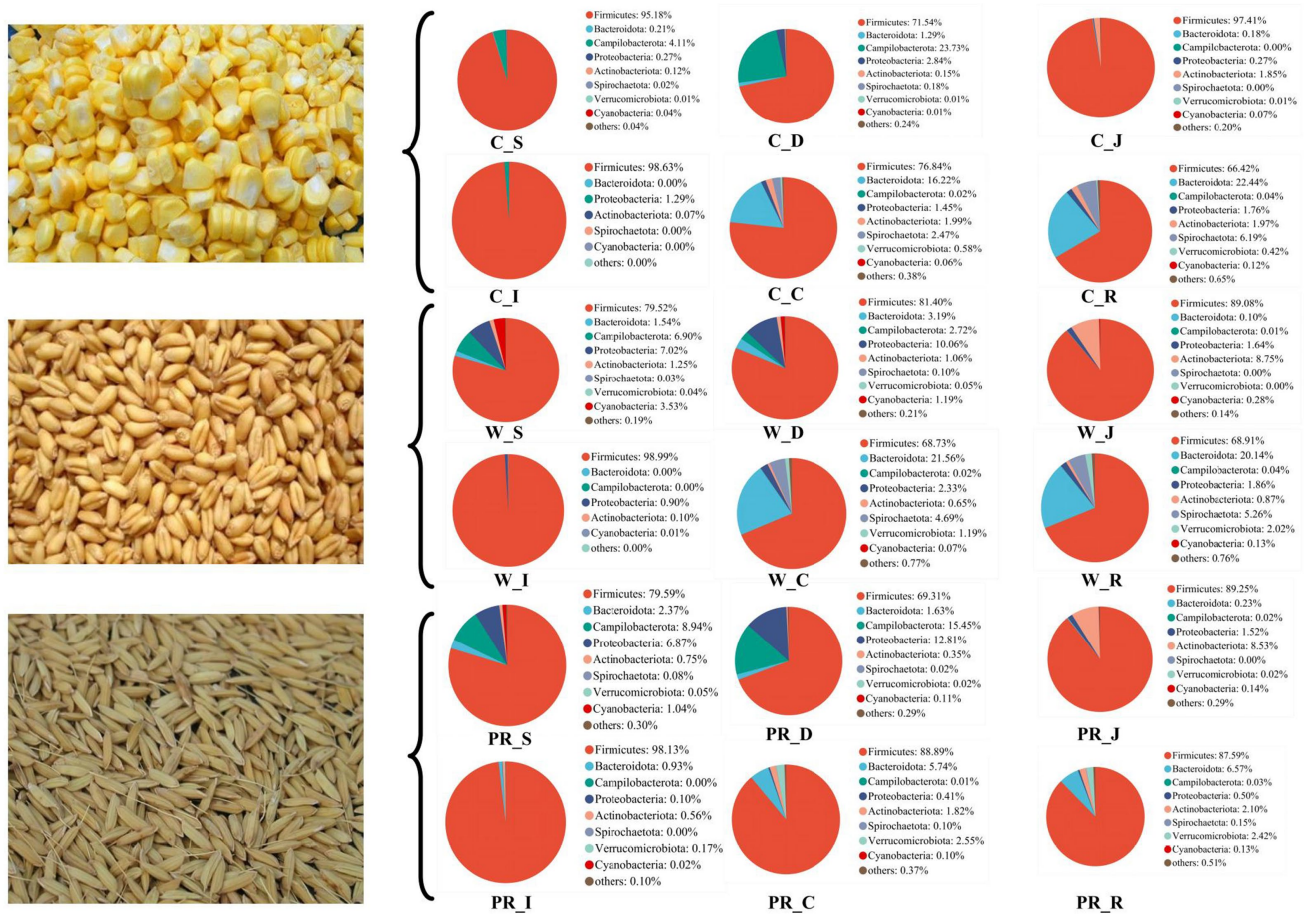
Composition of microbiota in the digesta of pigs fed with corn, wheat, and paddy rice diets based on the phylum level. C, corn; W, wheat; PR, paddy rice; S, stomach; D, duodenum; J, jejunum; I, ileum; C, colon; R, rectum.

**Figure 7:**
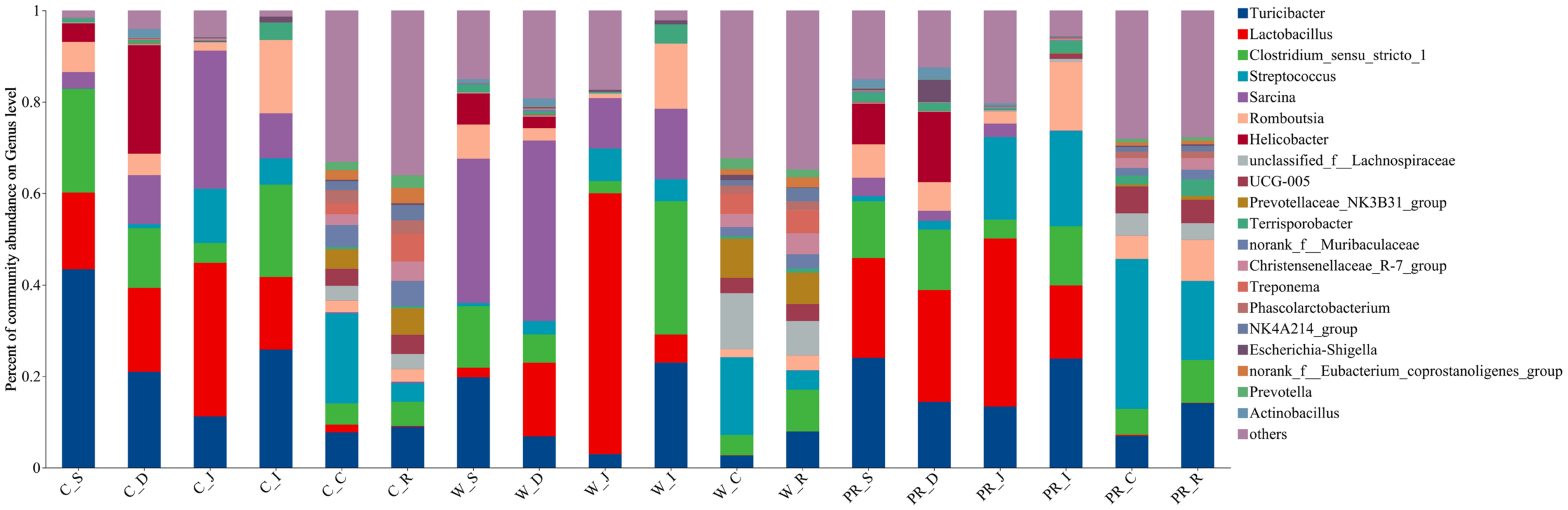
Composition of microbiota in the digesta of pigs fed with corn, wheat, and paddy rice diets based on the genus level. C, corn; W, wheat; PR, paddy rice; S, stomach; D, duodenum; J, jejunum; I, ileum; C, colon; R, rectum.

**Figure 8:**
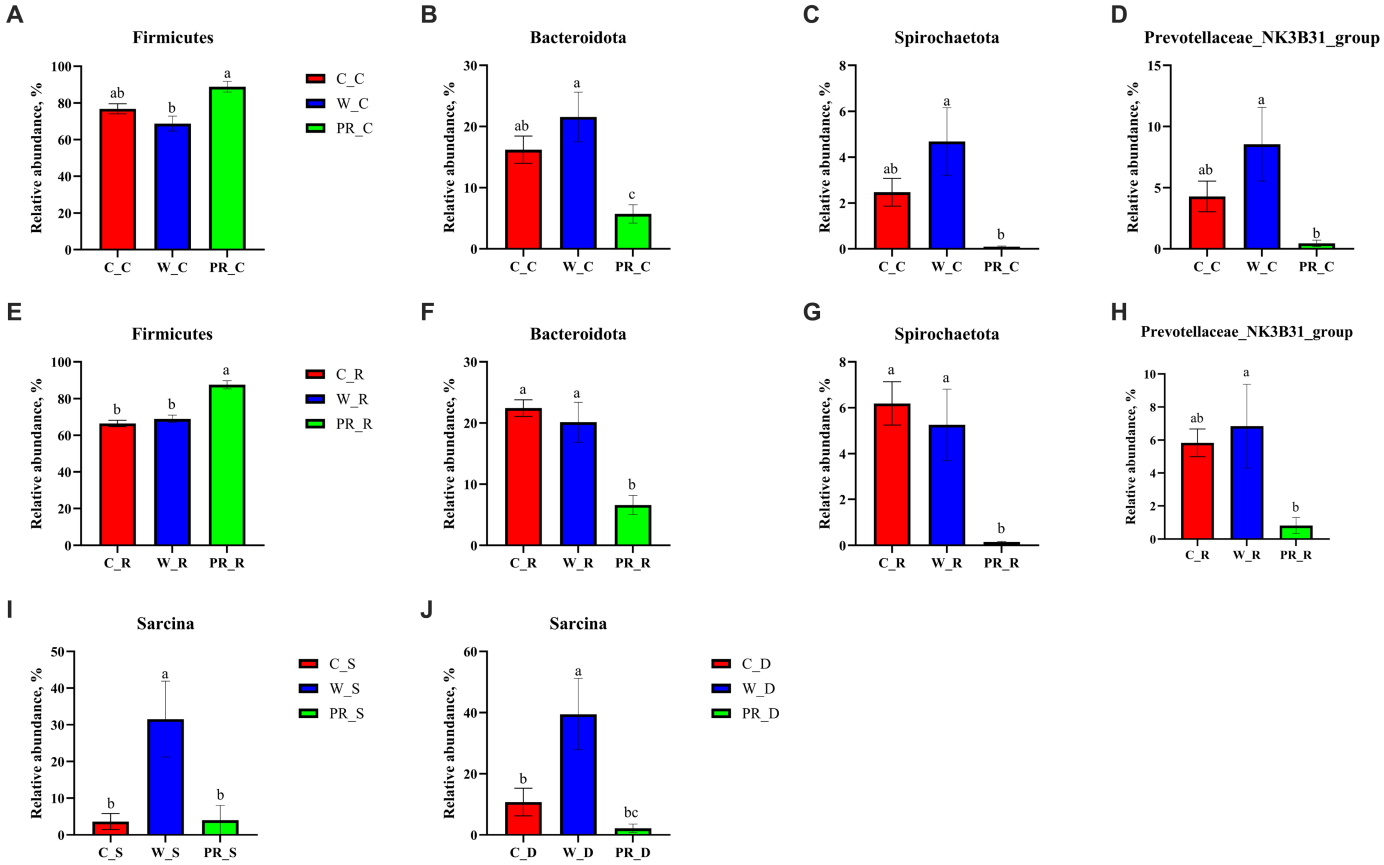
Comparative analysis of microbiota in the stomach **(I)**, duodenal **(J)**, colonic **(A–D)**, and rectal **(E–H)** digesta of adult pigs fed with corn, wheat, and paddy rice diets based on the phylum and the genus levels. All data were analyzed by the Kruskal–Wallis and Tukey-Kramer test and presented as the mean percentage of different bacteria (group = 3, *n* = 6 per treatment). The different letters mean *p* < 0.05. C, corn; W, wheat; PR, paddy rice; S, stomach; D, duodenum; C, colon; R, rectum.

As shown in Figure 6, Firmicutes was the most dominant phylum among dietary treatments. Campylobacterota was the second dominant phylum in the C_S and C_D groups (4.11 and 23.73%, respectively), while Proteobacteria was the second dominant phylum in the W_D group (10.06%). The W_S, PR_S, and PR_S groups have abundant Campylobacterota (6.90, 8.94, and 15.45%, respectively) and Proteobacteria (7.02, 6.87, and 12.81%, respectively). Actinobacteriota was the second dominant phylum in the W_J group (8.75%). Bacteroidota was the second dominant phylum in the C_C, W_C, PR_C, and PR_R groups (16.22, 21.56, 5.74, and 6.19%, respectively). Bacteroidota and Spirochaetota were the second (22.44 and 20.14%, respectively) and third (6.19 and 5.26%, respectively) dominant phyla in C_R and W_R, respectively. The top five genera in the relative abundances of all groups were *Turicibacter*, *Lactobacillus*, *Clostridium_sensu_stricto_1*, *Streptococcus,* and *Sarcina* (Figure 7). W_C and W_R groups had greater Bacteroidota, Spirochaetota, and *Prevotellaceae_NK3B31_group* abundances, but showed lower Firmicutes abundance than the PR_C and PR_R groups, respectively (*p* < 0.05, Figures 8A–H). The population of *Sarcina* in the W_S and W_D groups was the largest (*p* < 0.05, Figures 8I,J).

### 3.5 Cladogram of LEfSe from the phylum to the genus level

**Figure 9:**
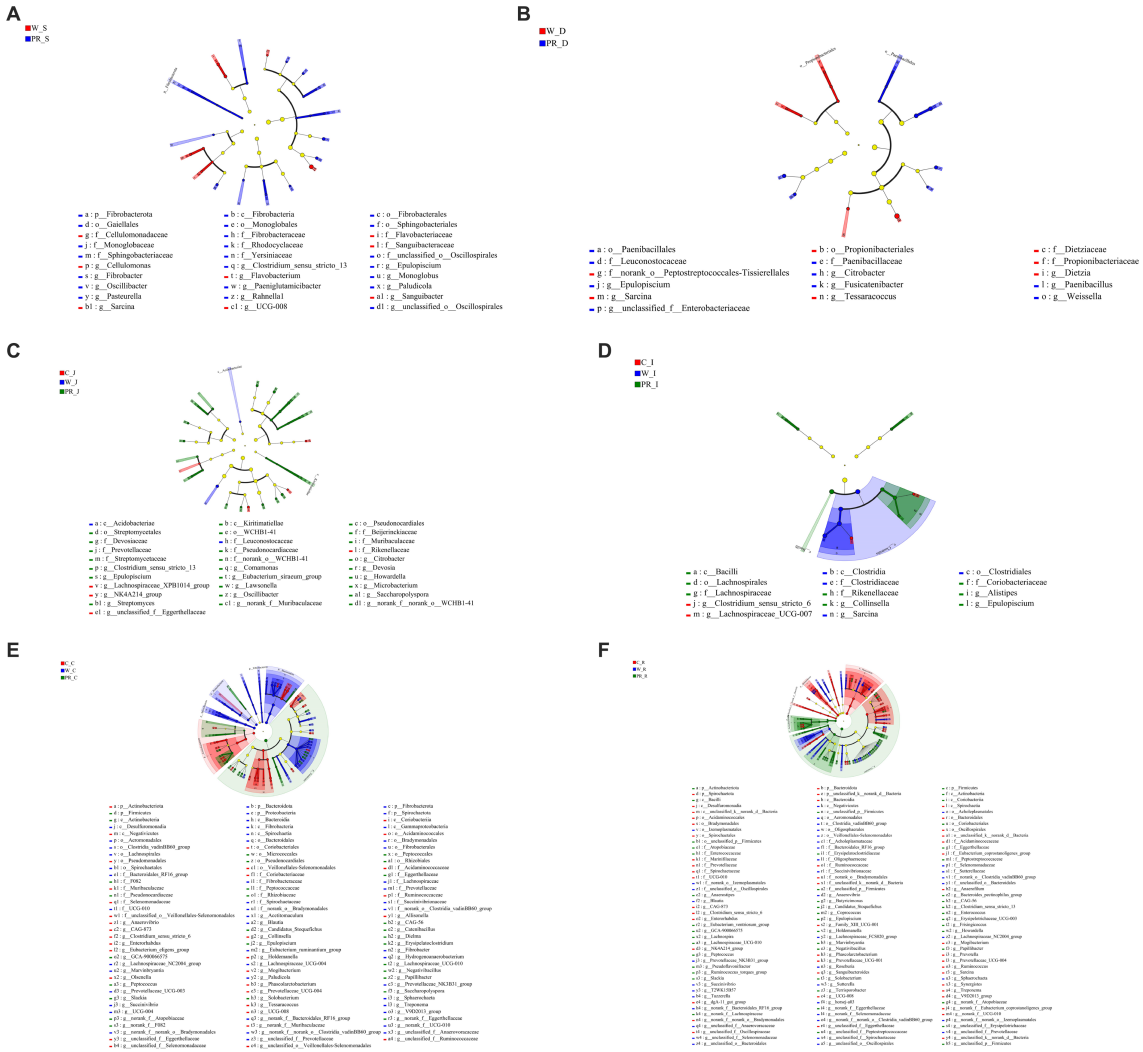
Cladogram of LEfSe demonstrates taxonomic profiling for the distinct bacteria in the stomach **(A)**, duodenal **(B)**, jejunal **(C)**, ileal **(D)**, colonic **(E)**, and rectal **(F)** digesta among three dietary treatments. C, corn; W, wheat; PR, paddy rice; S, stomach; D, duodenum; J, jejunum; I, ileum; C, colon; R, rectum.

From the phylum to the genus level, 8 taxa including *Sarcina* and 22 taxa, including Fibrobacterota, were significantly enriched in the W_S and PR_S groups, respectively (Figure 9A). Seven taxa, including *Propionibacteriales,* and nine taxa, including *paenibacillales*, were significantly enriched in the W_D and PR_D groups, respectively (Figure 9B). In the C_J, W_J, and PR_J groups, four specific enriched taxa, including *Acidobacteria*, two specific enriched taxa, including *NK4A214*, and 25 specific enriched taxa, including Kiritimatiellae, were detected, respectively (Figure 9C). Two (e.g., *Clostridium_sensu_strico_6*), four (e.g., *Clostridia*), and eight (e.g., *Bacilli*) taxa in the C_I, W_I, and PR_I groups were significantly enriched, respectively (Figure 9D). In total, 32 taxa, including Actinobacteriota, were significantly enriched in the C_C group, while 47 taxa, including Spirochaetota, Proteobacteria, Fibrobacterota, and Bacteroidota were significantly enriched in the W_C group, and 28 taxa, including Firmicutes, were significantly enriched in the PR_C group, respectively (Figure 9E). A total of 47 taxa represented by Spirochaetota and Bacteroidota were significantly enriched in the C_R group, while 39 taxa, including *Prevotellaceae_NK3B31_group,* were enriched in the W_R group, and 46 taxa, including Firmicutes and Actinobacteriota, were enriched in the PR_R group, respectively (Figure 9F).

### 3.6 SCFAs production

Acetate was the main SCFAs in the ileal digesta, while acetate, propionate, and butyrate were the dominant acids in the colonic and rectal digesta (Table 3). In ileal digesta, the contents of acetate and isovalerate in the W diet group were higher than in the C diet group (*p* < 0.01). The concentrations of propionate and total SCFAs in the W groups were higher than in other dietary groups (*p* < 0.01 and *p* < 0.05, respectively). The concentration of isobutyrate in the PR diet group was the lowest (*p* < 0.01). In the colonic digesta, the W diet group had greater acetate, propionate, butyrate, isobutyrate, isovalerate, and total SCFA contents compared with other dietary groups (*p* < 0.01 or *p* < 0.05). In the rectal digesta, the C and W diet groups showed higher acetate, propionate, and total SCFA contents than the PR group (*p* < 0.01). The W diet group had the highest concentration of butyrate (*p* < 0.05) and showed greater isobutyrate and isovalerate contents than other dietary groups (*p* < 0.01).

**Table 3:** Effects of different cereal grains on the concentrations of short-chain fatty acids in the intestine of pigs (μmol/g).

Items	Corn diet	Wheat diet	Paddy rice diet	SEM	*p*-value
Ileal digesta					
Acetate	4.96 ± 1.03^b^	20.54 ± 12.20^a^	12.06 ± 1.71^ab^	2.210	*p* < 0.01
Propionate	0.59 ± 0.21^b^	1.16 ± 0.30^a^	0.52 ± 0.40^b^	0.098	*p* < 0.01
Butyrate	0.83 ± 0.65	0.85 ± 0.48	0.85 ± 0.64	0.132	0.999
Isobutyrate	0.50 ± 0.01^a^	0.30 ± 0.00^b^	0.19 ± 0.04^c^	0.031	*p* < 0.01
Valerate	0.65 ± 0.09^a^	0.49 ± 0.00^b^	0.68 ± 0.10^a^	0.026	*p* < 0.01
Isovalerate	0.26 ± 0.08^b^	0.43 ± 0.06^a^	0.30 ± 0.18^ab^	0.032	*p* < 0.01
Total SCFAs	8.18 ± 1.88^b^	24.06 ± 12.37^a^	13.62 ± 3.94^b^	2.316	0.011
Colonic digesta					
Acetate	43.27 ± 15.78^b^	70.27 ± 11.34^a^	28.99 ± 4.95^c^	4.879	*p* < 0.01
Propionate	15.59 ± 5.31^b^	27.75 ± 5.25^a^	10.25 ± 2.82^b^	2.049	*p* < 0.01
Butyrate	11.43 ± 4.01^b^	28.32 ± 2.47^a^	6.79 ± 2.83^c^	2.351	*p* < 0.01
Isobutyrate	0.47 ± 0.01^c^	0.73 ± 0.13^a^	0.60 ± 0.04^b^	0.031	*p* < 0.01
Valerate	2.63 ± 2.05	2.03 ± 0.40	1.16 ± 0.36	0.308	0.147
Isovalerate	1.18 ± 0.20^b^	1.82 ± 0.45^a^	1.41 ± 0.08^b^	0.090	0.028
Total SCFAs	71.79 ± 22.60^b^	134.62 ± 23.79^a^	49.20 ± 10.07^b^	9.802	*p* < 0.01
Rectal digesta					
Acetate	35.38 ± 2.30^a^	38.62 ± 4.72^a^	22.43 ± 3.31^b^	1.873	*p* < 0.01
Propionate	13.36 ± 3.11^a^	14.31 ± 4.48^a^	6.09 ± 0.98^b^	1.138	*p* < 0.01
Butyrate	7.88 ± 4.19^ab^	13.68 ± 8.24^a^	3.49 ± 1.36^b^	1.565	0.023
Isobutyrate	0.49 ± 0.10^b^	0.91 ± 0.08^a^	0.48 ± 0.07^b^	0.052	*p* < 0.01
Valerate	1.16 ± 0.20	1.53 ± 0.40	1.16 ± 0.34	0.084	0.103
Isovalerate	1.42 ± 0.32^b^	2.36 ± 0.14^a^	1.26 ± 0.22^b^	0.129	*p* < 0.01
Total SCFAs	57.47 ± 5.10^b^	70.59 ± 16.85^a^	35.08 ± 5.31^c^	4.262	*p* < 0.01

The results were presented as mean values ± standard deviation (*n* = 6 per treatment). Means in each line with different letters differs significantly (*p* < 0.05).

### 3.7 Correlation analysis between the relative abundance of microbial community and SCFAs production

**Figure 10:**
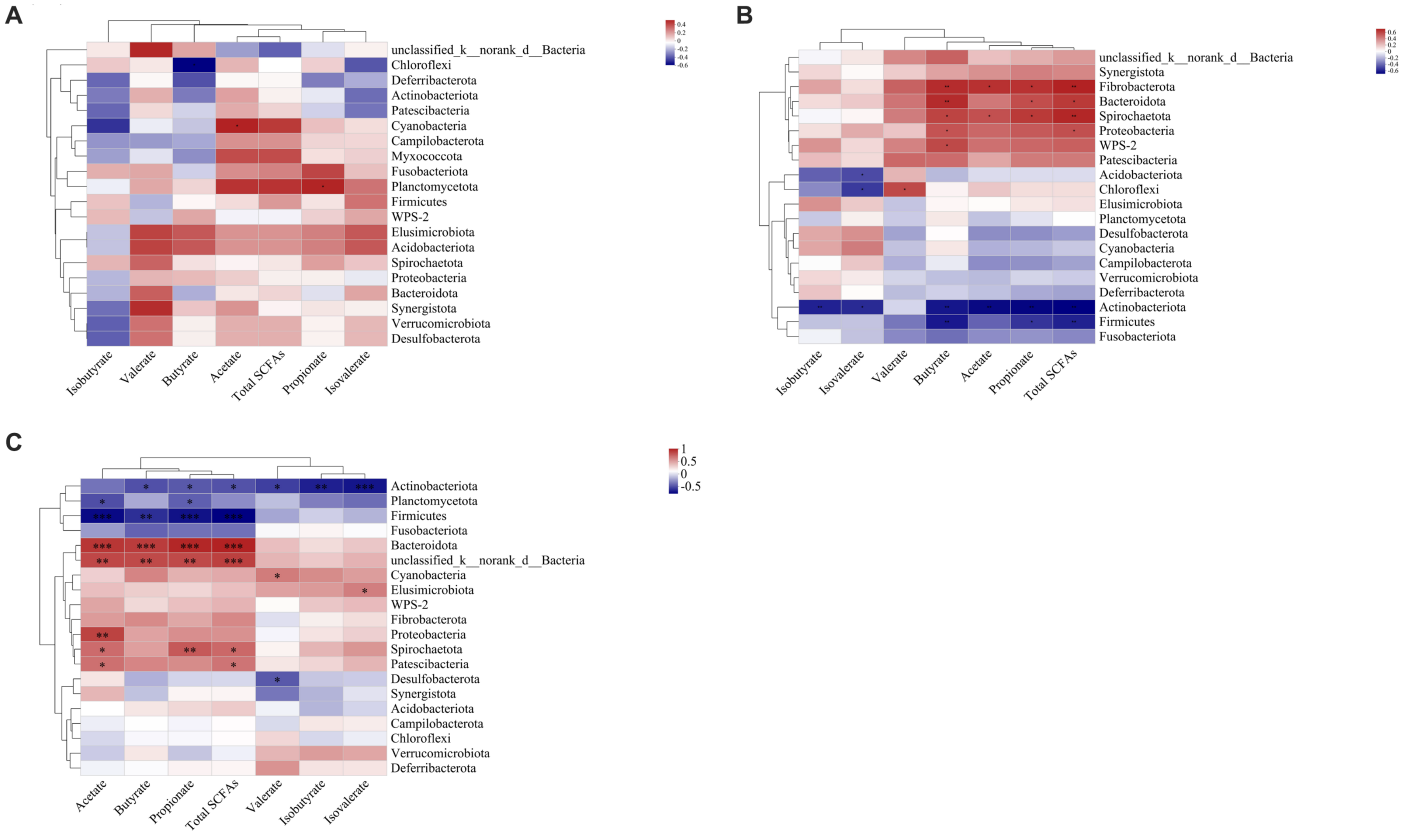
Correlation heatmaps between the short-chain fatty acid (SCFA) concentrations and the relative abundances of the top 20 phyla in the ileal **(A)**, colonic **(B)**, and rectal **(C)** digesta. Correlation is indicated by a color gradient from blue to red based on Spearman’s correlation coefficients. The asterisk indicates a significant correlation between two variables, *p < 0.05, **p < 0.01, ***p < 0.001.

**Figure 11:**
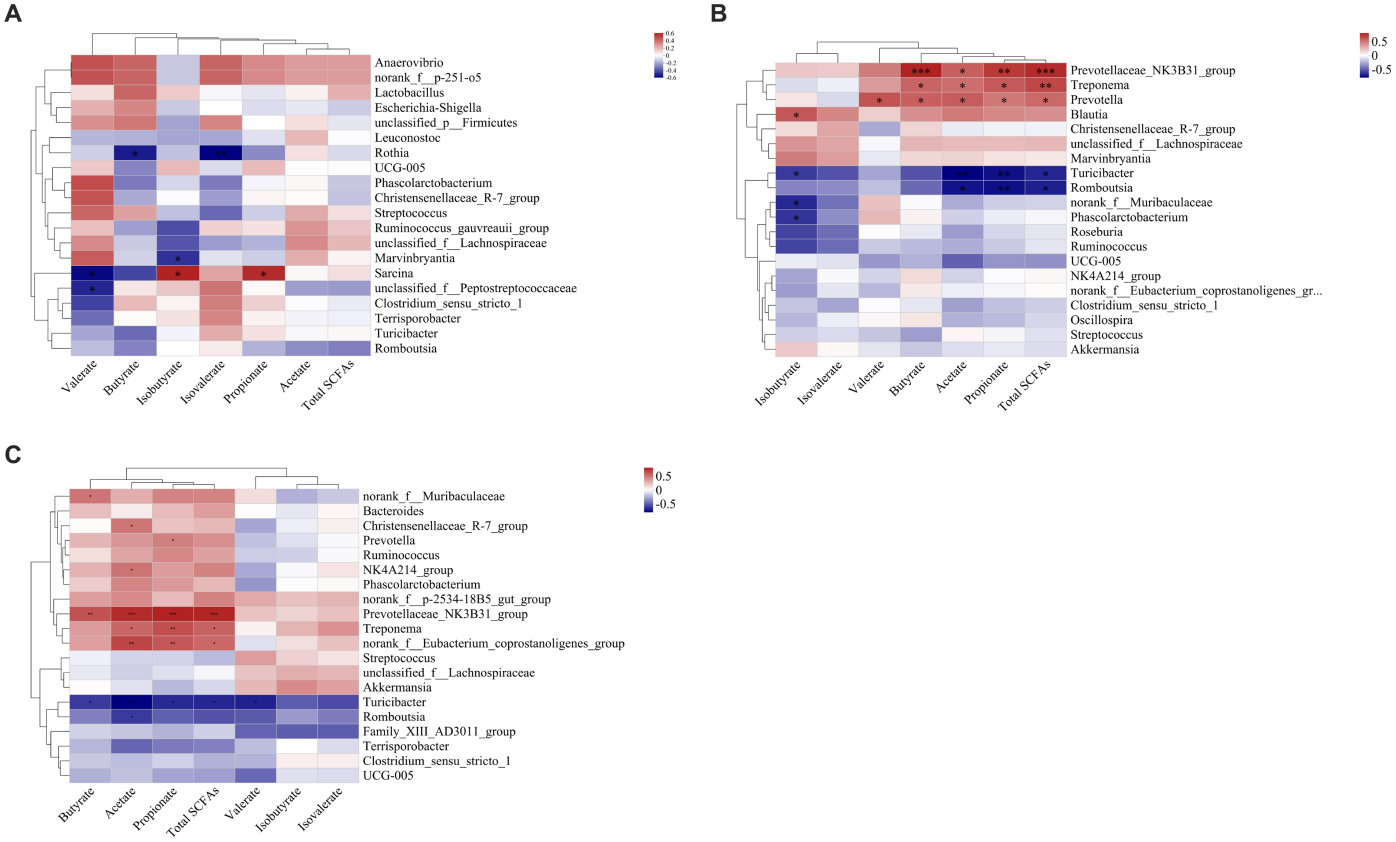
Correlation heatmaps between the short-chain fatty acid (SCFA) concentrations and the relative abundances of the top 20 genera in the ileal **(A)**, colonic **(B)**, and rectal **(C)** digesta. Correlation is indicated by a color gradient from blue to red based on Spearman’s correlation coefficients. The asterisk indicates a significant correlation between two variables, *p < 0.05, **p < 0.01, ***p < 0.001.

In the ileal digesta, Planctomycetota and *Sarcina* were positively correlated with propionate (*p* < 0.05, Figures 10A, 11A). In the colonic digesta, Fibrobacterota, Spirochaetota, *Prevotellaceae_NK3B31_group*, *Treponema,* and *Prevotella* showed positive correlations with acetate, propionate, butyrate, and total SCFAs (*p* < 0.05 or *p* < 0.01 or *p* < 0.001, Figures. 10B, 11B). Bacteroidota was positively correlated with propionate, butyrate, and total SCFAs, while Firmicutes was on the contrary (*p* < 0.05 or *p* < 0.01, Figure 10B). In the rectal digesta, Bacteroidota and *Prevotellaceae_NK3B31_group* showed strong positive correlations with acetate, propionate, butyrate, and total SCFAs, while Firmicutes was on the contrary (*p* < 0.01 or *p* < 0.001, Figures 10C, 11C). Spirochaetota and *Treponema* were positively correlated with acetate, propionate, and total SCFAs (*p* < 0.05 or *p* < 0.01, Figures 10C, 11C). Actinobacteriota in the colonic and rectal digesta were negatively correlated with acetate, propionate, butyrate, isobutyrate, isovalerate, and total SCFAs (*p* < 0.05 or *p* < 0.01 or *p* < 0.001, Figures 10B,C).

## 4 Discussion

### 4.1 α- and β-diversity of the samples

In this study, a greater number of unique OTUs (Supplementary Figure 1) and higher Shannon, Ace, and Chao1 indices were often observed in the colon and rectum compared with other digestive parts (Table 2, Supplementary Figures 2, 3). These indicate that the microbial communities in the colonic and rectal digesta samples had greater species richness and compositional diversity (Shannon, 1948; Simpson, 1949; Chao, 1984). Interestingly, we found that it was the digestive parts rather than dietary treatment that had a highly significant effect on the α-diversity indices of the samples (Table 2), which is similar to the previous study (Li et al., 2023). The PcoA results showed that the composition of microbial communities from the stomach to the ileum was approximate, but differed significantly from that of the colon and rectum (Figure 2A). These results were expected and consistent with the previous studies (Liu et al., 2019; Zhao et al., 2015). Anatomically, the duodenum, jejunum, and ileum belong to the small intestine, while the colon and rectum belong to the large intestine. The small intestine primarily relies on enzymes to digest most of the starch, protein, and fat in feed. However, the large intestine is the main site for the fermentation of DF, a small amount of protein and peptides produced by microorganisms. Therefore, different digestive patterns determine that the distribution of microorganisms will inevitably be segmented at the boundary between the small and large intestines (DiBaise et al., 2008; Liu et al., 2019; Zhao et al., 2015). In addition, compared with the large intestine, the pH value in the stomach is lower, the oxygen content in the stomach and small intestine (e.g., duodenum and jejunum) is higher, with more antimicrobials, faster peristalsis speed, and shorter transit time of digesta (Kelly et al., 2017). These factors are not conducive to the colonization of a large number of microorganisms. Therefore, there are only 10^3^–10^4^ bacteria/ml in the stomach and small intestine contents, mainly acid-tolerant lactobacilli and streptococci (Hao and Lee, 2004). As the oxygen concentration from the small intestine to the large intestine gradually decreases, the intestine peristalsis speed slows down, the pH value gradually increases, making it easier for microorganisms to colonize, and the number of microbial communities becomes more abundant and diverse (Hao and Lee, 2004). The colon also becomes the main site for microbial colonization, and a large number of specialized anaerobic bacteria adapted to this environmental condition, such as *Prevotellaceae*, which is intolerant to oxygen, gradually occupy a dominant position (Kelly et al., 2017). In summary, based on the different digestion patterns and physiological characteristics between the small intestine and the large intestine, it is reasonable to observe significant differences in the quantity and diversity of microbial communities between the anterior and posterior intestinal segments.

The composition of microbial communities in the colonic and rectal digesta also differed among three dietary treatment groups (Figures 2F,G), which is similar to the previous findings (Nielsen et al., 2014; Burbach et al., 2017; Li et al., 2023). This may be attributed to the difference in fermentation substrates in the large intestine. The responses of the microbiota to dietary intervention can be rapid and are also sensitive to structural changes in fermentation substrates (Turnbaugh et al., 2009; David et al., 2014; Hamaker and Tuncil, 2014; Fouhse et al., 2017). For each whole grain used in this experiment, due to its unique composition matrix, it affects the fluidity and availability of fermentation substrates entering the large intestine, thereby manipulating the microbial composition (Hamaker and Tuncil, 2014; Fouhse et al., 2017). Several previous studies have confirmed these viewpoints. It has been reported that a barley-based diet has a higher concentration of non-starch polysaccharides (NSP) and better water solubility than a W-based diet. Therefore, it increased the number of Firmicutes and decreased the number of Proteobacteria in the feces of pigs. Burbach et al. (2017) and Ellner et al. (2022) suggested that due to the higher cellulose and lower CP contents in rye than in triticale, cellulolytic species of *Clostridium_sensu_stricto* dominated in the ileum and feces of pigs fed with a rye-based diet, while the *Clostridium XI* level in the rye-based diet group was lower than that in the triticale-based diet group. Fouhse et al. (2017) reported that a large number of *Phascolarctobacterium* and *Oscillospira* were observed in the feces of pigs fed with a low-fermentability barley diet and hard red spring W diet, and speculated that this may be related to the high ADF content in these two diets. Snart et al. (2006) found that pigs consuming an oat-based diet showed significantly greater increases in Bifidobacteria compared with pigs consuming a barley-based diet, which may be related to the different structures and molecular weights of β-glucans and other polysaccharides, as well as differences in non-DF components. Similarly, the composition and structure of fermentation substrates entering the hindgut (e.g., cellulose, hemicellulose, lignin, and RS), vary greatly among C, W, and PR. This will inevitably change the composition of microbial communities and fermentation patterns in the hindgut (Bach-Knudsen, 2014; NRC, 2012; Cervantes-Pahm et al., 2014; Menkovska et al., 2017; Li et al., 2020; Kaur et al., 2021).

### 4.2 Differences in the microbial community among six digestive parts

The relative abundance of Firmicutes was dominant in six digestive parts (Figure 3), which is in agreement with previous findings (Gao et al., 2019a; Gao et al., 2019b; Zhao et al., 2019; Li et al., 2023). Firmicutes are believed to be involved in fat metabolism and energy absorption processes in the host (Zhao et al., 2015; Gao et al., 2019a). Some previous studies found that the Firmicutes level in the large intestine was greater than that in the small intestine and inferred that the large intestine might undertake more tasks of fat deposition compared to the small intestine (Mao et al., 2015; Zhao et al., 2015; Liu et al., 2018). However, our results do not concord with this inference (Figure 5A). In fact, Gao et al. (2019b) also suggested that the above inference might not be correct because they observed that there was no difference in the relative abundance of Firmicutes between the cecum and the small intestine of pigs. In this study, we observed that the Bacteroidota was mainly distributed in the colonic and rectal digesta (Figure 5B). Bacteroidota is rich in carbohydrate-active enzyme (CAZyme) genes, which can degrade plant cell walls and produce acetate and propionate in the large intestine (Morrison and Preston, 2016; Liu et al., 2021). Therefore, our result was as expected and close to the previous studies (Looft et al., 2014; Crespo-Piazuelo et al., 2018; Gao et al., 2019b). Campylobacterota is considered to be one of the important pathogens causing diarrhea in piglets after weaning (Adhikari et al., 2019). The increase of Proteobacteria abundance can be considered as one of the potential features of gut dysbiosis (Shin et al., 2015). Fortunately, compared to other groups, only the stomach and duodenum groups showed greater Campylobacterota and Proteobacteria abundances (Figures 5C,D).

On the genus level, we found that *Lactobacillus* was mainly distributed in the jejunum (Figure 5F), which is similar to the previous findings (Crespo-Piazuelo et al., 2018; Gao et al., 2019b). *Lactobacillus* is the most common probiotic, which contributes greatly to preventing potential infections and maintaining host intestinal health (Che et al., 2014; Ahn et al., 2018). Previous studies have shown that *Lactobacillus* was the major amylolytic genus (Pandey et al., 2023). Therefore, it was not surprising that abundant *Lactobacillus* appeared in the jejunum. Interestingly, we found that *Turicibacter* and *Clostridium_sensu_stricto_1* showed similar spatial regularities in the GIT, especially their high abundances in the ileum (Figures 5E,G), which is similar to some previous studies (Crespo-Piazuelo et al., 2018; Liu et al., 2019; Zhao et al., 2019). Some previous studies indicated that *Turicibacter* had good adaptability to an ileal environment with 5% oxygen content (Hillman et al., 1993; Cuiv et al., 2011; Looft et al., 2014). Meanwhile, a previous study has confirmed that the ileal effluent from ileostomy patients showed a high relative abundance of *Clostridium cluster I* (Zoetendal et al., 2012). Therefore, our results may be explained by the special environment of the ileum and the installation of the T-cannula. Currently, a higher relative abundance of *Streptococcus* in the colon was found Some members of Streptococcus are known for their high virulence (Spellerberg and Brandt, 2015) (Figure 5H), which may be due to its involvement in the fermentation of cereal grains through the Wood–Ljungdahl pathway (Koh et al., 2016). *Sarcina* has been demonstrated as a causative organism in the abomasa bloat and death of livestock (DeBey et al., 1996; Sopha et al., 2015). Crespo-Piazuelo et al. (2018) found that *Sarcina* was the relatively abundant genus in the duodenum, jejunum, and ileum of pigs, which is consistent with our results (Figure 5I).

In summary, the relative abundance of microbial communities from the stomach to the rectum was dynamically changing. Among them, some results can be explained by the decrease in oxygen concentration from the small intestine to the large intestine (Bergey et al., 2001; Albenberg et al., 2014; Donaldson et al., 2016). For example, we observed that aerobic bacteria or facultative anaerobes, such as Campylobacterota, Proteobacteria, and *Lactobacillus* in the foregut, were gradually replaced by anaerobic Bacteroidota and partially anaerobic *Streptococcus* in the hindgut (Figures 5B–D,F,H), which is strong evidence.

### 4.3 Differences in the microbial community among dietary treatment

Various grains contain different contents of macronutrients, organic, and inorganic micronutrients. This directly determines different fermentation substrates for intestinal microbiota and profoundly influences the relative abundances of specific dominant bacterial groups (Scott et al., 2008; Walker et al., 2011; Power et al., 2014; Gidley, 2023). In the current study, PR_C and PR_R groups had greater Firmicutes abundance than W_C and W_R groups, respectively (Figures 8A,E), suggesting that fat metabolism may be more vigorous in the hindgut of adult pigs fed with the PR diet (Li et al., 2023). However, the lower Bacteroidota abundance in the PR_C and PR_R groups may be associated with the high levels of ADF, *CF*, and IDF in the PR diet. These components are difficult to ferment due to their poor water solubility, which may inhibit the colonization of Bacteroidota (Urriola et al., 2010; Navarro et al., 2019a). The PR_C and PR_R groups showed lower Spirochaetota abundance than W_C and W_R, respectively (Figures 8C,G), indicating that the hindgut flora of pigs fed with PR may be more balanced (Litvak et al., 2017). This result may be explained by the different structure of arabinoxylan (AX) between W and PR. AX is one of the main components of NSP in cereal grains (Bach-Knudsen et al., 2017). AX in PR is located in the pericarp or testa with abundant branched chains, and the ratio of arabinose to xylose (A:X) is higher than that of W (0.8 versus 0.5–0.7; Zhang et al., 2015). Therefore, it is difficult to depolymerize. On the one hand, this ensures that the carbohydrates in PR are still slowly fermented in the distal part of the colon (Zhang et al., 2015; Bach-Knudsen et al., 2017). On the other hand, this will help reduce protein fermentation and the release of toxic substances and inhibit the proliferation of pathogenic microbes (Williams et al., 2005; Jha and Berrocoso, 2016). On the contrary, for W, AX is located in its aleurone layer with few branches and good water solubility (Zhang et al., 2015; Bach-Knudsen et al., 2017; Tiwari et al., 2019). These characteristics allow it to be rapidly fermented in the cecum and proximal colon (Bach-Knudsen et al., 2017). In addition, the protein level in the W diet is higher than that in the PR diet (11.12 versus 7.57%), thus there may be more undigested protein entering the hindgut. When the carbon source is gradually depleted in the hindgut, protein fermentation will occur in the distal of the colon, producing toxic metabolites such as ammonia, biogenic amines, hydrogen sulfide, indenol, and phenolic compounds (Rist et al., 2013; Molist et al., 2014; Giuberti et al., 2015; Pieper et al., 2016; Jha et al., 2019). This may provide a favorable growth environment for the colonization of potential pathogenic bacteria such as Spirochaetota (Shin et al., 2015).

*Prevotella* mainly uses carbohydrates as substrates and has the ability to hydrolyze protein, hemicellulose, and pectin (Varel and Yen, 1997; Burbach et al., 2017; Zhu et al., 2017; Han et al., 2023; Pandey et al., 2023). In this study, the cell wall of W contains abundant and highly soluble AX (Nortey et al., 2008; Bach-Knudsen, 2015; Jaworski et al., 2015), which may provide substrates for the colonization of *Prevotellaceae_NK3B31_group* in W_C and W_R groups (Figures 8D,H).

### 4.4 Effect of diet and digestive parts on SCFAs production

The DF, protein, and peptide in the cereal grain diet will be fermented by the microbiota in the cecum and colon (Macfarlane and Macfarlane, 2012). The main products are acetate, propionate, butyrate, H_2_, CH_4_, and CO_2_ (Cummings et al., 1987; Macfarlane and Macfarlane, 1993; Williams et al., 2001). The proportion of acetate can reach approximately two-thirds of the produced SCFAs (Williams et al., 2001; Lunn and Buttriss, 2007), which is consistent with our results (Table 3). Due to the large number of microbial communities that are highly active in the cecum and proximal colon, the concentrations of almost all kinds of SCFAs were increased in the colonic and rectal digesta (Louis et al., 2007). However, with the gradual consumption of fermentation substrates in the colon, a portion of SCFAs is utilized by colonocytes or absorbed into portal vein blood (Nielsen et al., 2014; Koh et al., 2016). These resulted in a decrease in the majority of SCFA contents in the rectum.

Our results showed that the W diet group had the highest concentrations of acetate, propionate, butyrate, and total SCFAs in the colonic and rectal digesta (Table 3). This means that microorganisms have a stronger fermentation effect on the W diet. Similarly, a previous study reported that whole-W diets exhibited higher SCFA concentrations in the cecum and colon of rats compared to the rice diet (Han et al., 2018). AX is the main type of NSP in W, with a high content (5.85–6.74%), less branched chains, and good water solubility, which makes it easy to be rapidly fermented (Zhang et al., 2015; Weiss et al., 2016; Bach-Knudsen et al., 2017; Navarro et al., 2019b). On the contrary, PR is difficult to ferment because of high IDF content, heavily branched AX, and the poor water solubility of β-glucan and lignin (Bach-Knudsen, 2014; Zhang et al., 2015; Tiwari et al., 2019). Isobutyrate and isovalerate are mainly formed by the metabolism of branched-chain amino acids such as valine, leucine, and isoleucine (Macfarlane et al., 1992). For the W diet group, we found that isobutyrate and isovalerate contents were the highest in the colonic and rectal digesta (Table 3). These results reveal that stronger protein fermentation appears in the hindgut of adult pigs fed a W diet.

### 4.5 Effect of microbial communities on SCFAs production

SCFAs are mainly the result of carbohydrate fermentation by specific microbial communities through different metabolic pathways (Gong et al., 2018). *Bacteroides* spp. and *Prevotella* spp. may convert pyruvate to acetate through the acetyl-CoA or the Wood–Ljungdahl pathway (Ragsdale and Pierce, 2008; Koh et al., 2016). Thus, in this study, we observed the significant positive relationships between Bacteroidota*, Prevotellaceae_NK3B31_group,* and acetate in the colon and rectum (Figures 10B,C, 11B,C). Propionate can be formed by some members of Bacteroidota via the succinate pathway (Scott et al., 2006; Reichardt et al., 2014). Therefore, we found that propionate was significantly positively correlated with Bacteroidota in the colon and rectum (Figures 10B,C), which is in line with our expectations. Butyrate is the preferred metabolic substrate and the main energy source of colon cells (Gardiner et al., 2020; Han et al., 2023). Butyrate is formed mainly through the phosphotransbutyrylase and butyrate kinase pathways, the butyryl-CoA: acetyl-CoA transferase pathway, and the lysine pathway (Duncan et al., 2002; Louis et al., 2004; Vital et al., 2014). Previous studies have shown that butyrate-producing bacteria are mainly *Clostridium clusters* IV and XIVa (Louis and Flint, 2009). However, no similar results were obtained in this study. Interestingly, some flora in the colon and rectum that were positively correlated with butyrate tend to be positively associated with acetate (Figures 10B,C, 11B,C). It has been shown that the presence of acetate results in the production of butyrate (Diez-Gonzalez et al., 1999). Based on this, we speculate that Fibrobacterota, Bacteroidota, Spirochaetota, *Prevotella_NK3B31_group,* and *Treponema* may have the ability to convert acetate to butyrate. Fibrobacterota in the colon was positively correlated with acetate, propionate, butyrate, and total SCFAs (Figure 10B), which is expected because Fibrobacterota can degrade cellulose and convert it to various SCFAs (Miron and Ben-Ghedalia, 1993).

A previous review summarized that Firmicutes and Actinobacteriota are important SCFA-producing bacteria (Gong et al., 2018). However, we found that Firmicutes and Actinobacteriota in the colon and rectum were negatively correlated with multiple SCFAs, while Spirochaetota, Proteobacteria, and *Treponema* were in contrast (Figures 10B,C, 11B,C). In general, higher concentrations of SCFAs will lead to a more acidic environment in the hindgut, which may inhibit the survival of potential pathogens such as Spirochaetota and Proteobacteria. Indeed, our results contradict it, which requires further experiments to explore the mechanism of this phenomenon in an adult pig model.

## 5 Conclusion

The diversity of microbial communities in the colon and rectum was similar but markedly different from that in the stomach, duodenum, jejunum, and ileum. From the stomach to the rectum, the evolution from aerobic bacteria and facultative anaerobes to anaerobes was observed. The W diet was more conducive to the colonization of Bacteroidota and *Prevotellaceae_NK3B31_group* that mainly used carbohydrates in the hindgut, however, a greater abundance of potential pathogenic bacteria was found. Meanwhile, the W diet showed higher concentrations of all SCFAs in the hindgut compared to the PR diet. These findings reveal the spatial variation regularities of GIT microbiota in the adult pig model, suggesting that W has better fermentability than C and PR, and provide new insights for GIT microbiota and metabolites responses to cereal grains diets.

## Data Availability

The data presented in the study are deposited into the Sequence Read Archive (SRA) database (https://www.ncbi.nlm.nih.gov/sra), under accession number PRJNA1091723 (https://www.ncbi.nlm.nih.gov/bioproject/PRJNA1091723).
