# Supplementary figures and images for: Gastrointestinal microbiota and metabolites responses to dietary cereal grains in an adult pig model
Source: Front Microbiol. 2024 Sep 17;15:1442077. doi: 10.3389/fmicb.2024.1442077 (PMC11442370; doi:10.3389/fmicb.2024.1442077)

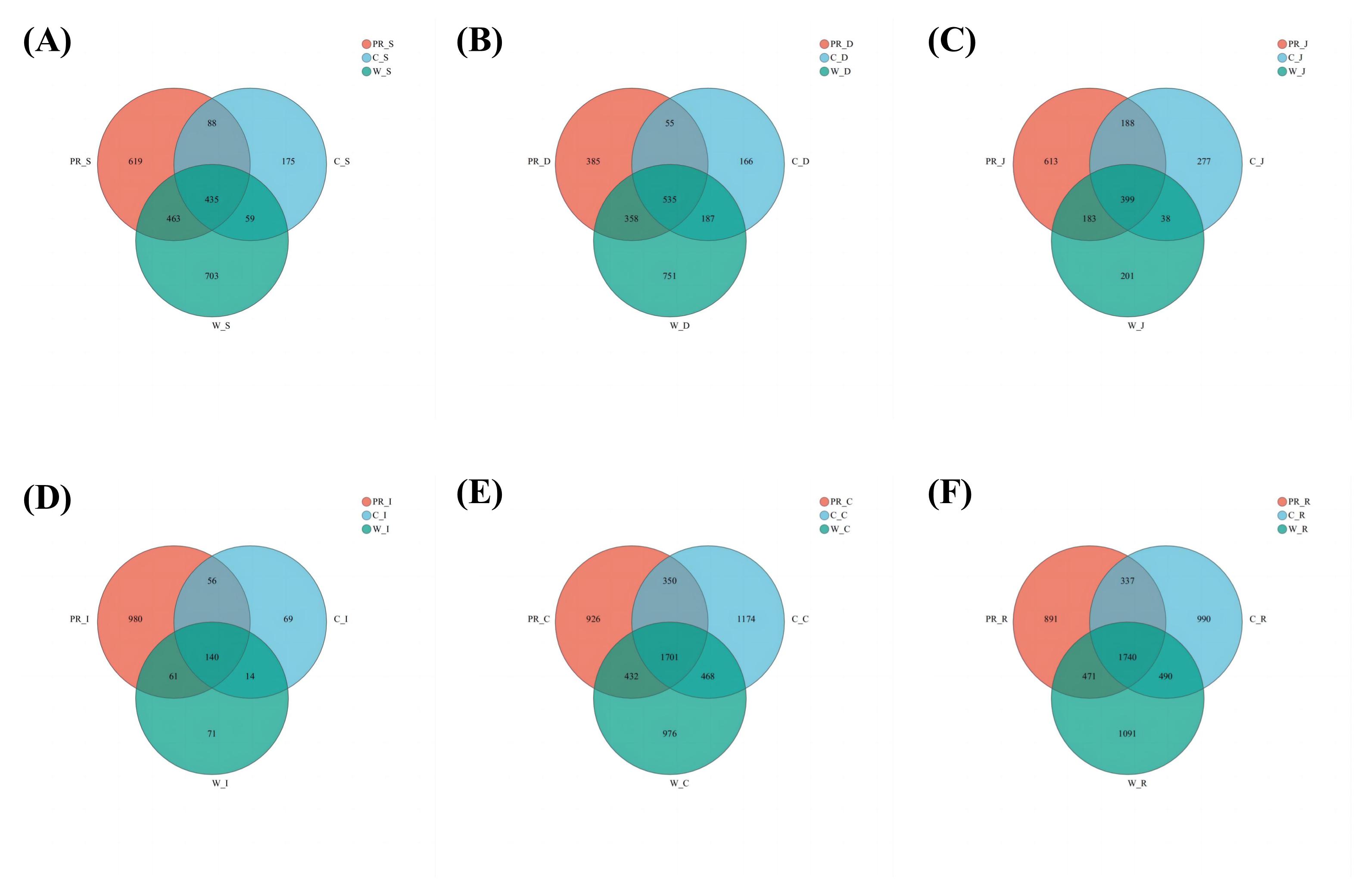

Supplement: Supplementary file 1 [file Image_1.JPEG]

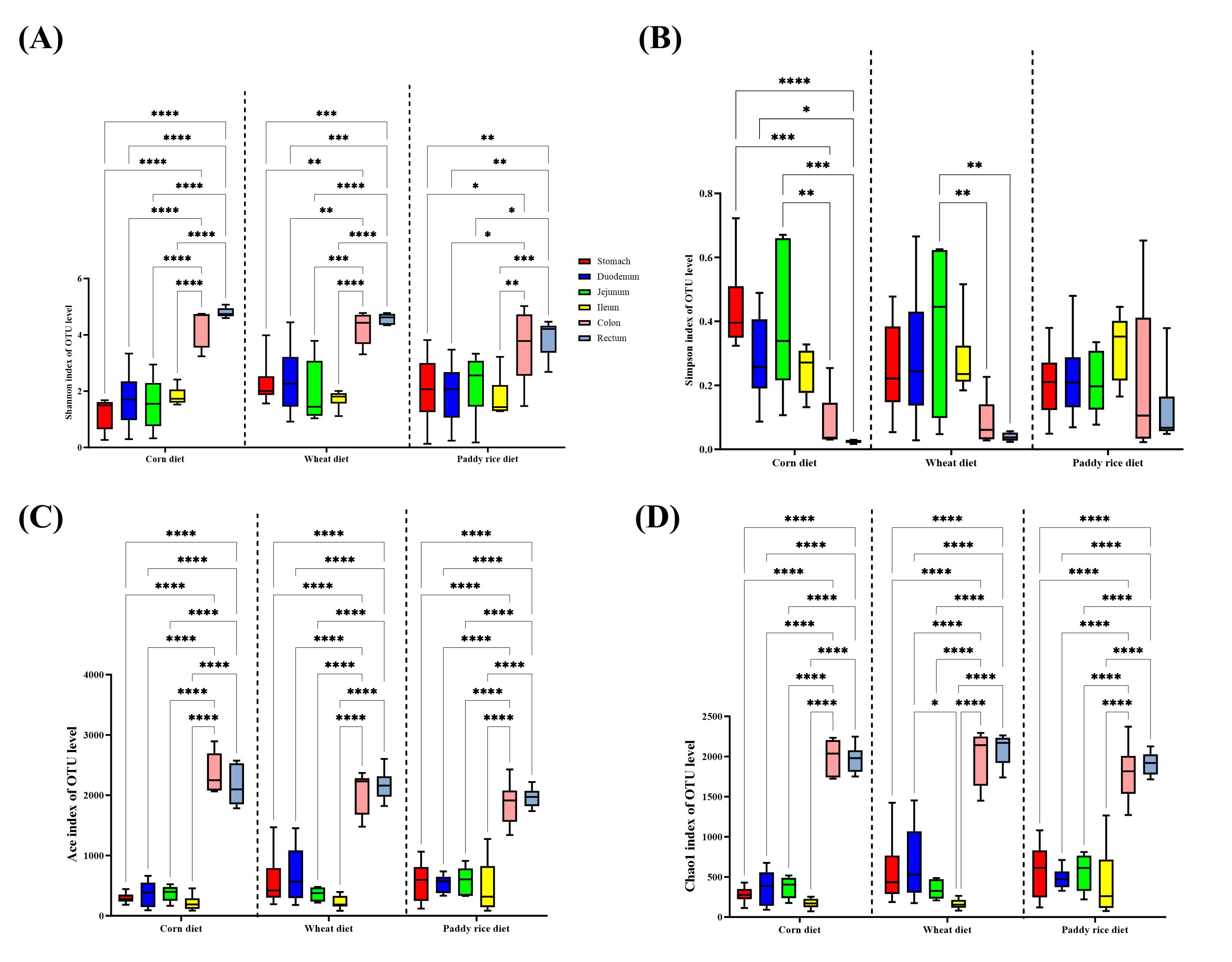

Supplement: Supplementary file 3 [file Image_3.JPEG]
